# Inadequate sleep hygiene as a key factor in poor sleep quality in systemic sclerosis: an observational, cross-sectional study

**DOI:** 10.1007/s00296-025-05794-7

**Published:** 2025-01-31

**Authors:** Neslihan Gokcen, Andac Komac, Fatma Tuncer Kuru, Ozlem Ozdemir Isik, Duygu Temiz Karadag, Ayten Yazici, Ayse Cefle

**Affiliations:** Faculty of Medicine, Department of Internal Medicine, Division of Rheumatology, Kocaeli University, Kocaeli, Turkey

**Keywords:** Depression, Rheumatoid arthritis, Sleep hygiene, Sleep quality, Systemic sclerosis

## Abstract

This study aims to investigate the relationship between sleep hygiene and sleep quality in patients with systemic sclerosis (SSc) and to compare the sleep hygiene and sleep quality outcomes across three distinct groups: SSc patients, rheumatoid arthritis (RA) patients, and healthy controls (HC). This study employed an observational, cross-sectional, and parallel group design. SSc-related and RA-related variables, depression and anxiety were assessed. Physical function and quality of life, pain and fatigue of SSc patients were also evaluated. Sleep quality using the Pittsburg Sleep Quality Index (PSQI) and sleep hygiene using the Sleep Hygiene Index (SHI) were evaluated for all participants. Linear regression analysis was performed to show the relationship between the SHI scores and the other variables. Total PSQI and SHI scores were found to be significantly higher in SSc patients than in RA patients and HC. Fatigue, smoking, all SF-36 domains, depression and anxiety scores were associated with SHI scores in SSc patients. In the univariate logistic regression analysis, SSc patients exhibited 4.50 times higher odds (95% CI 2.165–9.353, *p* < 0.001) of experiencing poor sleep than RA patients and HC. In SSc patients, for every incremental increase in SHI score, the odds of poor sleep quality were 1.15 times higher (95% CI 1.093–1.220, *p* < 0.001). Sleep hygiene and sleep quality exhibit a more pronounced deterioration in SSc patients. Inadequate sleep hygiene is associated with compromised sleep quality in SSc. Therefore, improving sleep hygiene practices may be a key strategy to enhance the overall sleep quality in this population.

## Introduction

Systemic sclerosis (SSc) is a rare autoimmune disorder typified by cutaneous and internal organ fibrosis, as well as vasculopathy [1]. In addition to cutaneous manifestations, the involvement of several organs, particularly the heart and lung, is frequently observed in SSc patients [2]. Rheumatoid arthritis (RA) is a persistent systemic autoimmune disorder primarily characterized by inflammatory arthritis, typically affecting the small joints of the hands and feet. Analogously to SSc, the involvement of several organs can be illustrated in those patients [3]. Both SSc and RA have the potential to result in substantial disability and mortality.

Sleep disorders represent a widespread issue within the general populace, with an estimated incidence rate of approximately 30% [4]. However, the prevalence of sleep disturbances is significantly higher among individuals with rheumatic diseases, exceeding 75% [5]. Decreased sleep quality occurs as an inevitable consequence of the sleep disorders experienced by rheumatic patients, including patients with systemic sclerosis (SSc) and rheumatoid arthritis (RA). In SSc, the underlying etiologies for sleep disturbances and compromised sleep quality exhibit variations; however, the most prominently recognized causative factor is arthritis, accounting for sleep issues in 72% of rheumatic patients aged 55 and older [4, 6, 7]. Furthermore, pain, fatigue, high disease severity, skin deformities, functional status, esophageal involvement, dyspnea, pulmonary hypertension, restless legs syndrome, and depression have been identified as factors associated with sleep disturbances in patients with SSc [6, 8–10]. Similarly, sleep problems have been also observed in patients with RA. Recent studies indicate that factors such as disease activity, disease duration, pain, inflammation, comorbidities (such as mood and cognitive disorders) and medications (such as antidepressants, benzodiazepines, hypnotics, opioids, etc.) are associated with the development of sleep issues in RA patients [7, 11–13].

Hence, the existing literature contains numerous studies examining the frequency of sleep-related problems in SSc and RA, elucidating their underlying causes, and assessing overall sleep quality [4, 5, 14–16]. Sleep hygiene, which refers to healthy sleep habits encompassing lifestyle, environmental, and behavioral strategies, plays a pivotal role in sleep quality [17]. Therefore, the optimization of sleep hygiene practices appears a readily accessible avenue for mitigating sleep disturbances [18, 19]. In line with that, numerous studies show that enhancing sleep hygiene practices improves sleep quality in various populations, such as students, older adults, and athletes [17]. However, interestingly, as far as we know, there is no data regarding the sleep hygiene and its impact on the sleep quality of patients with SSc. Accordingly, the primary aim of this study is to evaluate the impact of sleep hygiene on sleep quality and to examine its association with clinical parameters in patients with SSc. The secondary objective is to compare the sleep hygiene and sleep quality results across three groups: SSc patients, RA patients, and healthy controls.

## Materials and methods

### Study design and study population

This study was designed as an observational, cross-sectional, and parallel group study. Seventy patients with SSc who fulfilled the 2013 American College of Rheumatology (ACR)/European League Against Rheumatism (EULAR) classification criteria for systemic sclerosis, 70 age-matched disease controls (RA patients who met 2010 ACR/EULAR classification criteria), and 70 age-matched healthy controls (HC) were included in the study [20, 21]. Only female participants were selected. SSc patients were excluded based on the following criteria: (i) classification under the New York Heart Association (NYHA) Functional Class IV, indicating symptoms of heart failure at rest, (ii) the presence of active digital ulcers, (iii) concurrent acute arthritis, myositis, psychological or neurological disorders, and (iv) any recent changes in treatment regimen or disease status within the preceding three-month period. RA patients were excluded as follows: (i) the presence of severe joint deformities significantly limiting their range of motion, (ii) the occurrence of acute arthritis, (iii) the presence of psychological and neurological disorders, and (iv) alterations in treatment regimen and disease status within the preceding three-month period.

The study was conducted in compliance with the Declaration of Helsinki. Written informed consent was obtained from all participant. The study protocol and consent documentation were approved by the Kocaeli University Ethics Committee (approval number = GOKAEK-2019/7.10. 2019/137).

### Evaluation of study population

The demographic and clinical characteristics included age, body mass index (BMI) (kg/m^2^), physical activity/amount of exercise, current smoking status, alcohol consumption, disease duration, organ involvement, SSc-related variables (Raynaud’s phenomenon, digital pitting scars, arthralgia, joint contracture, sclerodactyly, calcinosis, color change, and telangiectasia), RA-related variables (Raynaud’s phenomenon, arthralgia, joint deformities, and rheumatoid vasculitis), medical treatment, and comorbidities. The socio-economic status, such as years of education and monthly household income, were also recorded.

Laboratory results such as erythrocyte sedimentation rate (ESR) (mm/h), and C-reactive protein (CRP) (mg/dl), rheumatoid factor (RF), anti-citrullinated protein antibody (ACPA), anti-nuclear antibody (ANA), anti-Scl-70 antibody, anti-centromere antibody, complement levels (C3 and C4) were recorded.

### Medical and SSc-related variables

Disease duration was defined as the period starting from the initial manifestation of non-Raynaud’s phenomenon symptoms. The definition of LeRoy et al. was used to classify patients into limited cutaneous SSc (lcSSc) and diffuse cutaneous SSc (dcSSc) subsets [22]. SSc-related variables and organ involvement were documented after the comprehensive review of medical records and the inquiries conducted with the patients. Accordingly, interstitial lung disease (ILD), pulmonary hypertension (PH), scleroderma renal crisis, and gastrointestinal involvement were noted. Skin involvement was assessed using the Modified Rodnan Skin Score (mRSS) [23]. Disease activity and severity were evaluated by Valentini disease activity index (VDAI) and Medsger disease severity scale (MDSS) [24, 25].

Nailfold capillaroscopy (NFC) was performed by a single experienced specialist (DTK) using a digital microscope (Dino-Lite Capillary-Scope 200, Naarden, Netherlands) and a related software program (The Dino-Capture v2.0 software from AnMo Electronics Corp., Taiwan). SSc patients were evaluated under an appropriate temperature of 20–25°. Eight fingers were assessed with 200× magnification, capturing at least two adjacent fields of 1 mm after applying immersion oil to the nailfold epidermis. NFC characteristics were classified into five distinct patterns, categorized as the non-scleroderma pattern (comprising normal and non-specific abnormalities) and the scleroderma pattern (encompassing early, active, and late patterns) [26].

### Medical and RA-related variables

Disease duration was defined as the time elapsed from the onset of arthritis. RA-related variables and organ involvement were documented by searching medical databases and questioning patients. Accordingly, extraarticular manifestations such as pulmonary and cardiac involvement, subcutaneous nodules, rheumatoid vasculitis, and neuromuscular involvement were recorded. Furthermore, the presence of gastroesophageal reflux leading to sleep disturbance was assessed in all participants. The Disease Activity Score 28-joint C-reactive protein (DAS28-CRP) was used to determine disease activity [27, 28].

#### Physical function and quality of life

Health Assessment Questionnaire-Disability Index (HAQ-DI) and Short-Form 36 (SF-36) were used to evaluate physical functioning and quality of life in SSc patients. Only HAQ-DI was used in RA patients [29, 30].

Pain and fatigue levels were assessed by an 11-point pain numerical rating scale (NRS). The Numeric Rating Scale for pain (NRS-pain) enables the assessment of pain along a continuous scale, spanning from 0 (representing the absence of pain) to 10 (indicating severe pain). Similarly, the Numeric Rating Scale for fatigue (NRS-fatigue) facilitates the evaluation of fatigue levels on a scale ranging from 0 (indicating no fatigue) to 10 (representing the most extreme level of fatigue) [4, 31].

The physical activity and exercise levels of the participants were classified into four distinct groups, namely sedentary, light exercise, moderate exercise, and very/extremely active. Comorbidity was assessed with the Charlson Comorbidity Index (CCI). This index includes 19 items presenting different medical conditions. Each item is assigned a distinct score based on its diverse clinical weights, with higher scores displaying severe comorbid conditions [32]. Healthy controls (HC) underwent assessment of comorbidity using the CCI. HC with comorbidities that did not result in organ dysfunction, physical disability, or sleep disturbances were included in the study.

### Depression and anxiety

Beck Depression Inventory (BDI) and Beck Anxiety Inventory (BAI) were used to define the psychological status of the study population [33, 34].

### Sleep quality and sleep hygiene

Sleep quality of all participants was assessed using the Pittsburg Sleep Quality Index (PSQI). PSQI is a self-report instrument designed to assess sleep quality and disturbances experienced within the preceding month. This tool encompasses nineteen items distributed across seven components: sleep quality, sleep latency, sleep duration, habitual sleep efficiency, sleep disturbance, sleeping medication, and daytime dysfunction. Each component within the assessment is assigned a score ranging from 0 to 3. The cumulative global score encompasses the range from 0 to 21, with scores of 6 or higher indicating poor sleep quality [35].

Sleep hygiene was evaluated using the Sleep Hygiene Index (SHI), which is a reliable and valid self-administered questionnaire consisting of 13 inquiries. Each question is scored as 0 (never), 1 (rarely), 2 (sometimes), 3 (frequently), and 4 (always). The sum of scores ranges from 0 to 52, whereby higher scores denote a greater manifestation of behaviors that undermine sleep hygiene in the subjects [36].

### Statistical analysis

Power analysis was performed using the G*Power 3.1.9.4 software program. Considering the absence of prior information in this specific domain, the determination of the sample size in this study was based on general considerations rather than a pre-specified difference. Consequently, a sample size of 69 participants per group (totaling 207 subjects) was deemed necessary to achieve a statistical power of 90% in detecting a significant difference in SHI scores, assuming an alpha error of 0.05 and an effect size of 0.25. We thus included 210 patients in the study.

Statistical analyses were conducted utilizing the Statistical Package for the Social Sciences (SPSS), version 26 (IBM Inc., Armonk, NY, USA). Descriptive analyses were employed to examine the sociodemographic variables. The normality of the distribution of continuous variables was assessed using of the Shapiro-Wilk test. Non-parametric continuous variables were compared using the Mann-Whitney U and Kruskal-Wallis tests, while parametric continuous variables were compared using independent samples t-test and one-way Analysis of Variance (ANOVA). Categorical parameters were analyzed using the chi-squared test and Fisher’s exact test.

Linear regression analysis (simple linear regression, backward and stepwise multiple linear regression) was employed to define the most appropriate linear equation for characterizing the relationship between alterations in the SHI scores and variations in the other variables. In addition, this analysis was performed to understand the effect of one unit increased in SHI scores on PSQI subscales and total scores. The results were given as (coefficient value [95% CI], p value). Additionally, univariate and multivariate logistic regression analyses were conducted to ascertain the contributing factors for sleep quality.

Receiver Operating Characteristic (ROC) curve analysis was conducted to determine the cutoff value of the Sleep Hygiene Index (SHI) score, enabling the differentiation of subjects with poor sleep quality from those with good sleep quality. The optimal cut-point value in the ROC analysis was identified using the Index of Union method [37]. The Area Under the Curve (AUC) was categorized based on previously established thresholds: excellent (0.9-1.0), good (0.8–0.9), fair (0.7–0.8), poor (0.6–0.7), or fail (0.5–0.6) [38]. Statistical significance was defined as a p-value < 0.05.

## Results

**Fig. 1:**
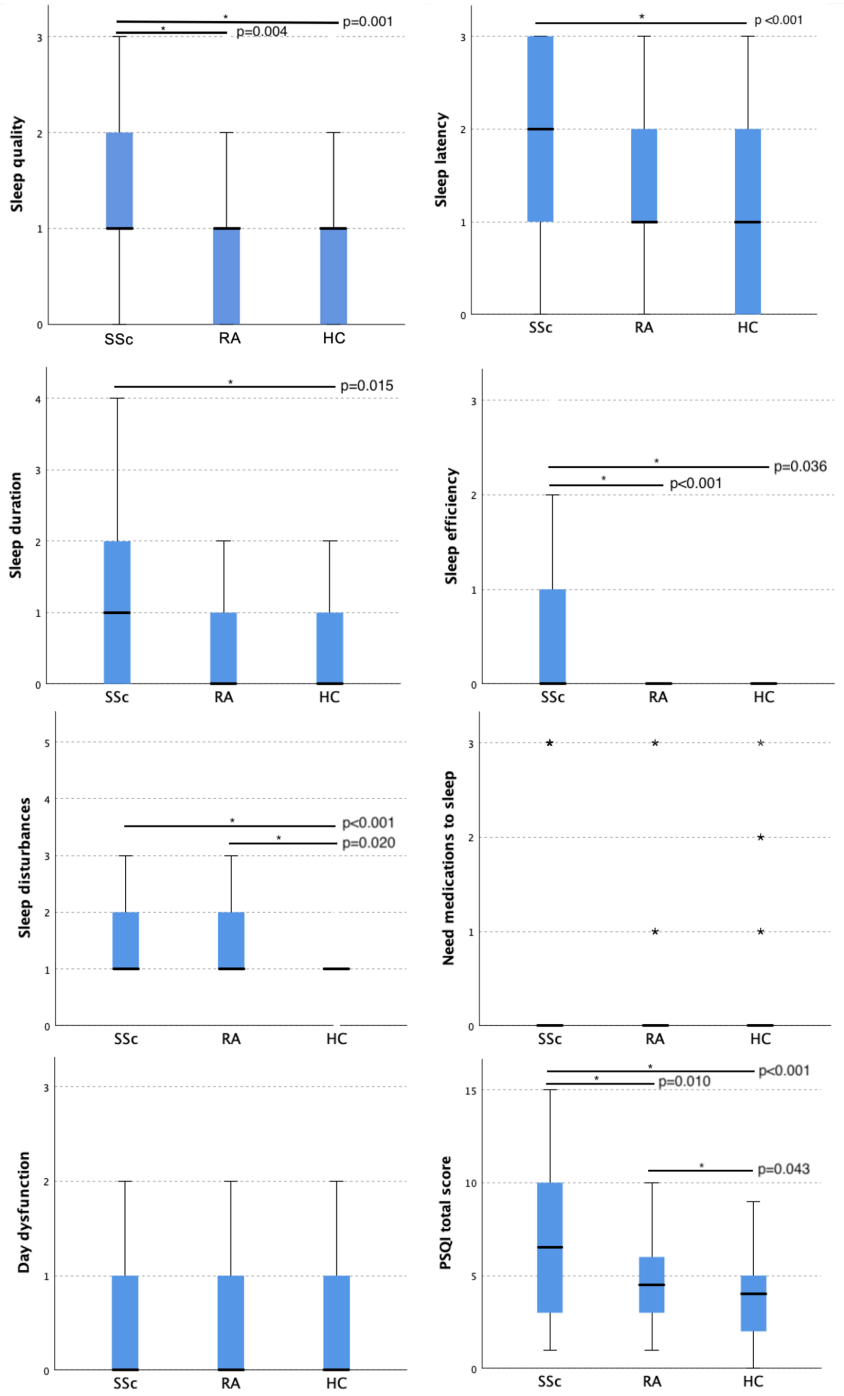
Pairwise comparisons of Pittsburgh Sleep Quality Index subscales and total scores between groups

The characteristics of the study groups (70 SSc patients, 70 RA patients, 70 HC) are given in Table 1. When the groups were compared, total PSQI and SHI scores were found to be significantly higher in SSc patients. Specifically, patients with SSc exhibited higher scores in various PSQI subscales, including sleep quality, sleep latency, sleep efficiency, and sleep disturbances. Figure 1 demonstrates pairwise comparisons of PSQI subscales and total scores between groups. Additionally, paired comparison of SHI showed that SSc patients had higher scores than RA patients (*p* < 0.001) and HC (*p* < 0.001). However, there were not any differences between RA patients and HC. Interestingly, poor sleepers (with total PSQI scores of 6 or higher) were more prevalent in SSc patients than in RA patients and HC (*p* < 0.001).

**Table 1:** Sociodemographic and clinical characteristics of the study population

Variables	SSc (*n* = 70)	RA (*n* = 70)	HC (*n* = 70)	*p*
Age (year)^a^	52.7 (46.3–60.0)	53.5 (46.5–63.0)	50.0 (41.8–58.0)	0.075
BMI (kg/m^2^)^b^	28.4±4.4	29.6±5.4	26.7±4.5	**0.002**
Education (year)^a^	5.0 (5.0–11.0)	5.0 (5.0-8.8)	11.0 (5.0-15.3)	**< 0.001**
Income (TL/month)^a^	17.000 (15.000-18.750)	15.900 (15.250-18.000)	18.000 (15.250-22.000)	**< 0.001**
CCI^a^	3.0 (2.0-3.3)	2.0 (1.0-2.3)	0 (0–1)	**< 0.001**
Current smoker^c^	12 (17.1)	15 (21.4)	11 (15.7)	0.659
GERD^c^	51 (72.9)	18 (25.7)	12 (17.1)	**< 0.001**
Diabetes Mellitus^c^	10 (14.3)	13 (18.6)	4 (5.7)	0.069
NRS-pain^a^	5.0 (3.0–6.0)	3.0 (2.0–4.0)	0 (0–1.0)	**< 0.001**
NRS-fatigue^a^	3.0 (2.0–5.0)	2.0 (1.0–4.0)	1.0 (0–3.0)	**< 0.001**
HAQ-DI^a^	3.0 (1.0-7.3)	4.0 (1.0–13.0)	0 (0)	**< 0.001**
BDI^a^	10.0 (6.0–15.0)	9.0 (4.0-14.3)	8.0 (3.8–11.0)	**0.019**
BAI^a^	12.5 (4.8–18.3)	7.5 (3.8–14.0)	8.0 (3.8–12.0)	**0.007**
PSQI^a^				
Sleep quality	1.0 (1.0–2.0)	1.0 (0–1.0)	1.0 (0–1.0)	**0.002**
Sleep latency	2.0 (1.0–3.0)	1.0 (0.8-2.0)	1.0 (0–2.0)	**0.002**
Sleep duration	1.0 (0–2.0)	0 (0–1.0)	0 (0–1.0)	0.049
Sleep efficiency	0 (0–1.0)	0 (0)	0 (0)	**0.001**
Sleep disturbances	1.0 (1.0–2.0)	1.0 (1.0–2.0)	1.0 (1.0–1.0)	**< 0.001**
Need medications to sleep	0 (0)	0 (0)	0 (0)	0.773
Day dysfunction	0 (0–1.0)	0 (0–1.0)	0 (0–1.0)	0.188
PSQI total score	6.5 (3.0–10.0)	4.5 (3.0-6.3)	4.0 (2.0–5.0)	**< 0.001**
Poor sleepers^c^	40 (57.1)	22 (31.4)	16 (22.9)	**< 0.001**
SHI^a^	14.0 (8.8–18.0)	8.5 (6.0–15.0)	8.5 (6.0–13.0)	**< 0.001**

BMI, Body Mass Index; CCI, Charlson Comorbidity Index; GERD, Gastroesophageal Reflux Disease; NRS, Numeric Rating Scale; HAQ-DI, Health Assessment Questionnaire-Disability Index; BDI, Beck Depression Inventory; BAI, Beck Anxiety Inventory; PSQI, Pittsburg Sleep Quality Index; SHI, Sleep Hygiene Index

^a^Values given as median (interquartile range), Kruskal Wallis test

^b^Values given as Mean ± standard deviation, One way ANOVA

^c^Values given as n (%), Chi-Square test

In the univariate logistic regression analysis, SSc patients exhibited 4.50 times higher odds (95% CI 2.165–9.353, *p* < 0.001) of experiencing poor sleep than RA patients and HC. After adjusting for age and smoking status in the multivariate logistic regression analysis, patients with SSc still demonstrated 4.61 times higher odds (95% CI 2.199–9.664, *p* < 0.001) of having poor sleep. Furthermore, for every incremental increase in SHI score, the odds of poor sleep quality were 1.15 times higher (95% CI 1.093–1.220, *p* < 0.001). Table 2 provides an overview of the findings derived from the logistic regression analysis performed exclusively on patients with SSc, offering insights into the factors associated with poor sleep quality. Accordingly, following adjustments for significant variables identified in the univariate analysis, each increased score in the BDI was associated with 1.17 times higher odds of poor sleep quality, while SSc patients with GERD showed 6.82 times higher odds of experiencing poor sleep.

**Table 2:** Univariate and Multivariate logistic regression analyses between good and poor sleepers in SSc patients

	Univariate logistic regression analysis		Multivariate logistic regression analysis			
			Model 1		Model 2	
	OR (95% CI)	p	OR (95% CI)	p	OR (95% CI)	p
Age (year)	0.972 (0.929–1.016)	0.202	0.987 (0.929–1.049)	0.675	-	-
NRS-pain	1.313 (1.025–1.684)	0.031	1.328 (0.947–1.863)	0.100	1.273 (0.958–1.692)	0.096
CCI	1.762 (1.015–3.058)	0.044	1.476 (0.642–3.395)	0.359	-	-
GERD	6.125 (1.882–19.934)	0.003	4.771 (1.032–22.050)	0.045	6.817 (1.747–26.609)	0.006
SF-36						
Social functioning	0.977 (0.956–0.999)	0.042	1.014 (0.980–1.049)	0.432	-	-
Role emotional	0.984 (0.972–0.997)	0.016	0.993 (0.976–1.010)	0.435	-	-
Mental health	0.956 (0.929–0.984)	0.002	0.995 (0.951–1.040)	0.819	-	-
BDI	1.181 (1.063–1.312)	0.002	1.122 (0.957–1.315)	0.155	1.172 (1.043–1.317)	0.007
BAI	1.099 (1.027–1.177)	0.006	1.031 (0.927–1.148)	0.574	-	-
SHI	1.136 (1.039–1.243)	0.005	1.045 (0.927–1.178)	0.473	-	-

NRS, Numeric Rating Scale; CCI, Charlson Comorbidity Index; GERD, gastroesophageal reflux disease; SF-36, Short Form 36; BDI, Beck Depression Inventory; BAI, Beck Anxiety Inventory

**Fig. 2:**
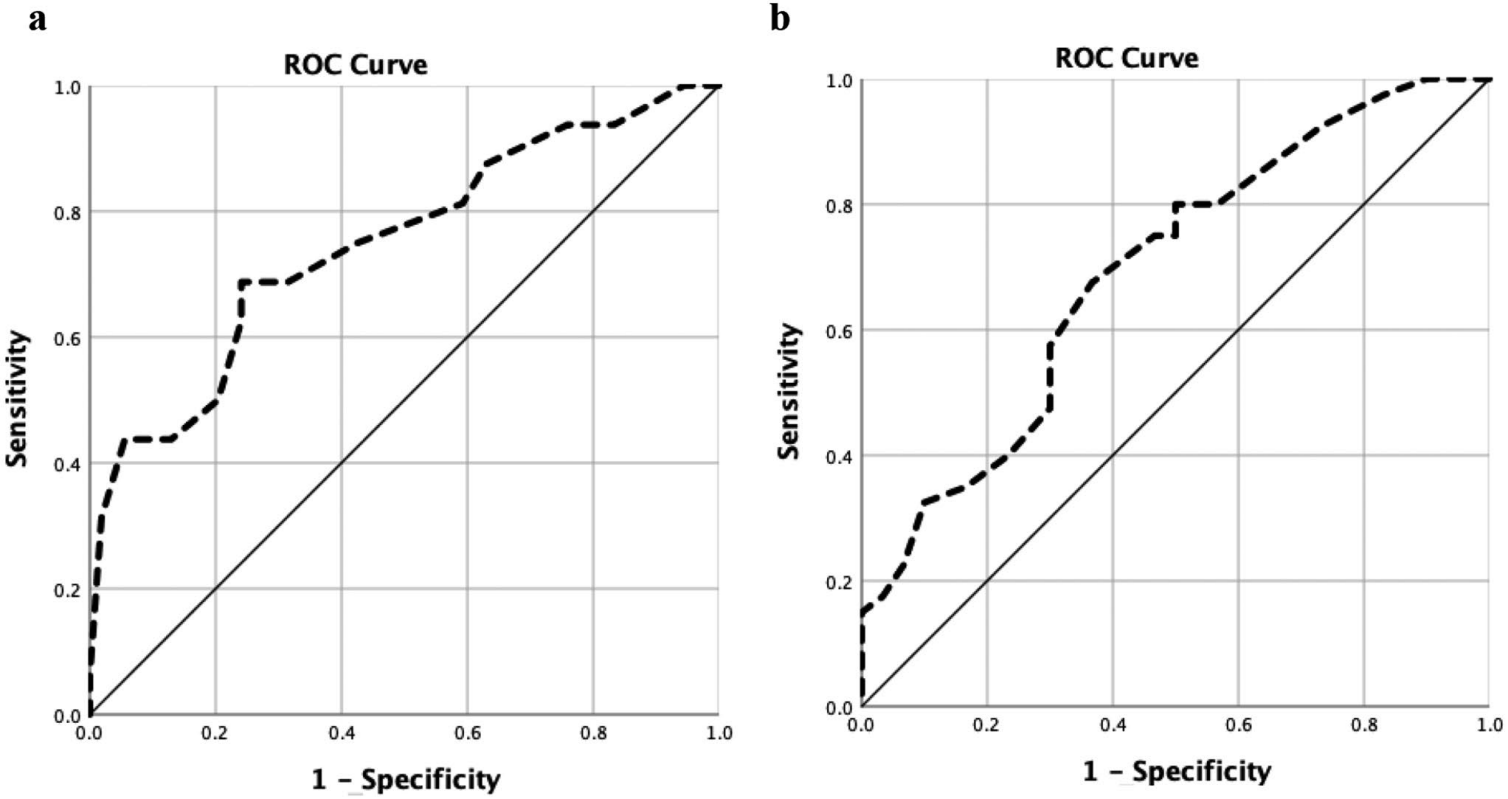
ROC curves of sleep hygiene index scores in determining poor sleepers in healthy controls (**a**) and systemic sclerosis patients (**b**)

In ROC analysis, the cut-off value of SHI scores to define HC with poor sleep was 8.5 with 75.0% sensitivity and 57.4% specificity (AUC = 0.75, 95% CI 0.60–0.89, *p* = 0.003). However, in the case of SSc patients with poor sleep, the cut-off value was determined to be 10.5 with a sensitivity of 80.0% and specificity of 50.0% (AUC = 0.69, 95% CI 0.57–0.82, *p* = 0.006). ROC analyses are shown in Fig. 2. Conversely, this analysis failed when applied to patients with RA.

Of 70 SSc patients, 58.6% (*n* = 41) were limited cutaneous SSc and 41.4% (*n* = 29) were diffuse cutaneous SSc. Most SSc patients (*n* = 62, 88.6%) were engaging in moderate exercise. Concerning organ involvement, 64.3% (*n* = 45) had interstitial lung disease (ILD), and 20.0% (*n* = 14) exhibited pulmonary hypertension (PH). Within SSc patients, it was observed that 50.0% (*n* = 35) manifested the presence of anti-centromere antibody, whereas 41.1% (*n* = 29) displayed the presence of anti-Scl-70 antibody. Table 3 presents a comparative analysis of clinical variables between SSc patients characterized by good sleep quality (PSQI < 6) and those exhibiting poor sleep quality (PSQI ≥ 6). Accordingly, in line with expectations, poor sleepers had a higher frequency of GERD and higher scores on the NRS-pain (*p* = 0.002 and *p* = 0.015, respectively). Moreover, depressive symptoms, anxiety, and worse sleep hygiene were prevalent in poor sleepers. However, contrary to initial expectations, it was observed that good sleepers exhibited higher BMI values and longer disease durations compared to poor sleepers (*p* = 0.016 and *p* = 0.014, respectively). Additionally, the frequency of several clinical manifestations, including Raynaud’s phenomenon, digital pitting scars, arthralgia, joint contracture, sclerodactyly, calcinosis, color change, and telangiectasia, was similar between the two groups.

**Table 3:** The comparison of clinical variables between SSc patients with good sleep quality and SSc patients with poor sleep quality

	Good Sleepers (PSQI < 6) (*n* = 30)	Poor sleepers (PSQI ≥ 6) (*n* = 40)	*p*
Age (year)^a^	56.0 (43.0–66.0)	52.0 (47.3–56.0)	0.086
BMI (kg/m^2^)^b^	29.8±4.4	27.3±4.0	**0.016**
Disease duration (year)^b^	10.8±6.6	7.4±4.7	**0.014**
lcSSc/dcSSc^c^	16 (53.3)/14 (46.7)	25 (62.5)/ 15 (37.5)	0.299
ILD^c^	18 (60.0)	27 (67.5)	0.616
PH^c^	6 (20.0)	8 (20.0)	1.000
GERD^c^	16 (53.3)	35 (87.5)	**0.002**
NRS-pain^a^	4.0	5.5	**0.015**
NRS-fatigue^a^	3.0	3.0	0.133
NFC findings			
Normal	3 (10.0)	7 (17.5)	
Non-specific abnormalities	1 (3.3)	4 (10.0)	
Early pattern	4 (13.3)	8 (20.0)	
Active pattern	18 (60.0)	19 (47.5)	
Late pattern	4 (13.3)	2 (5.0)	
ESR (mm/hour)^a^	16.5 (10.5–22.0)	12.0 (8.0–20.0)	0.094
CRP (mg/L)^a^	4.5 (3.1–9.1)	2.5 (1.1–4.6)	**0.004**
MRSS^a^	8.0 (4.0-13.3)	7.5 (3.3–11.8)	0.175
SF-36^a^			
Physical function	80.0 (58.8–90.0)	65.0 (50.0–80.0)	0.072
Role physical	62.5 (0-100.0)	25.0 (0–75.0)	0.141
Body pain	61.0 (48.8–75.0)	41.5 (34.0–61.0)	**0.044**
General health	47.0 (34.3–67.8)	50.0 (35.0–61.0)	0.868
Vitality	50.0 (38.8–66.3)	40.0 (25.0-53.8)	0.098
Social functioning	100.0 (62.0-100.0)	75.0 (53.0–87.0)	**0.013**
Role emotional	66.0 (33.0-100.0)	33.0 (0-100.0)	**0.016**
Mental health	70.0 (60.0–88.0)	54.0 (40.0–72.0)	**< 0.001**
HAQ-DI^a^	2.0 (1.0-5.3)	4.0 (1.3-8.0)	0.128
BDI^a^	7.5 (4.8–11.0)	12.0 (8.3–18.8)	**0.001**
BAI^a^	8.0 (3.0–14.0)	13.5 (8.0–22.0)	**0.003**
SHI^b^	11.2±5.9	15.5±5.7	**0.003**
MDSS^a^	5.0 (3.8-6.0)	4.5 (3.3-6.0)	0.995
VDAI^a^	1.0 (0.5–2.5)	2.0 (1.0-2.5)	0.381

BMI, Body Mass Index; lcSSc, limited cutaneous SSc; dcSSc, diffuse cutaneous SSc; ILD, interstitial lung disease; PH, pulmonary hypertension; GERD, Gastroesophageal Reflux Disease; ESR, Erythrocyte Sedimentation Rate; CRP, C-reactive protein; MRSS: Modified Rodnan Skin Score; SF-36, Short Form 36; HAQ-DI, The Health Assessment Questionnaire-Disability Index; BDI, Beck Depression Inventory; BAI, Beck Anxiety Inventory; MDSS, Medsger Disease Severity Scale; VDAI, Valentini Disease Activity Index

^a^Values given as median (interquartile range), Mann-Whitney U test

^b^Values given as Mean ± standard deviation, Independent samples t test

^c^Values given as n (%), Fisher’s exact test

In SSc patients, when using simple linear regression analysis to find the association between SHI scores (dependent variable) and other variables including fatigue, smoking cigarette, all SF-36 domains, BDI, and BAI scores were found to be associated with SHI scores. In backward multiple linear regression analysis, SF-36 mental health subscale was associated with SHI (Table 4). Furthermore, when SHI scores were regarded as an independent variable, each incremental increase of one unit in SHI scores corresponded to a deterioration in sleep quality (coefficient 0.035 [0.001, 0.070], *p* = 0.045), sleep latency (coefficient 0.079 [0.038, 0.120], *p* < 0.001), sleep duration (coefficient 0.059 [0.015, 0.103], *p* = 0.009), daytime dysfunction (coefficient 0.033 [0.001, 0.065], *p* = 0.043), and total scores on the PSQI (coefficient 0.216 [0.066, 0.365], *p* = 0.005).

**Table 4:** Simple and multiple linear regression analyses between Sleep Hygiene Index scores and other variables in SSc patients

	Simple Linear regression analysis		Multiple Linear regression analysis*	
	Coefficient (95% CI)	p	Coefficient (95% CI)	p
NRS-pain	0.702 (-0.003, 1.407)	0.051	-0.438 (-1.554, 0.677)	0.435
NRS-fatigue	0.890 (0.131, 1.649)	0.022	0.079 (-0.895, 1.053)	0.871
Smoking cigarette	4.353 (0.568, 8.139)	0.025	0.983 (-3.001, 4.968)	0.623
SF-36				
Physical function	-0.069 (-0.137, -0.002)	0.045	-0.011 (-0.093, 0.072)	0.796
Role physical	-0.038 (-0.073, -0.002)	0.038	0 (-0.048, 0.047)	0.983
Body pain	-0.090 (-0.151, -0.030)	0.004	-0.097 (-0.208, 0.015)	0.089
General health	-0.064 (-0.127, -0.001)	0.047	0.014 (-0.058, 0.085)	0.703
Vitality	-0.091 (-0.154, -0.029)	0.005	0.047 (-0.045, 0.139)	0.311
Social functioning	-0.082 (-0.138, -0.025)	0.005	-0.010 (-0.084, 0.064)	0.797
Role emotional	-0.047 (-0.083, -0.012)	0.010	-0.014 (-0.062, 0.034)	0.560
Mental health	-0.147 (-0.204, -0.089)	< 0.001	-0.146 (-0.243, -0.048)	0.004
BDI	0.348 (0.166, 0.530)	< 0.001	-0.018 (-0.323, 0.287)	0.907
BAI	0.225 (0.074, 0.376)	0.004	-0.003 (-0.203, 0.198)	0.979

NRS, Numeric Rating Scale; SF-36, Short Form 36; BDI, Beck Depression Inventory; BAI, Beck Anxiety Inventory

*Backward multiple linear regression analysis was used

## Discussion

The current study revealed that SSc patients exhibited lower sleep hygiene and worse sleep quality. In addition, we found that SSc patients with poor sleep had worse sleep hygiene than SSc patients with good sleep. From a different perspective, we observed that with each increase in SHI score, the likelihood of poor sleep quality in SSc patients was 1.15 times higher. In line with our study, numerous studies proved that SSc patients had poor sleep quality [6–9, 39–41]. A study evaluating sleep disruption in SSc indicated that polysomnographic evaluation of those patients contained fragmented sleep patterns characterized by reduced sleep efficiency and reduced rapid eye movement sleep, increased arousal index, and increased slow-wave sleep [39]. Another study investigating polysomnography findings of SSc patients demonstrated that the sleep duration, sleep quality, and rapid eye movement ratio of SSc patients decreased [42]. According to a Canadian National Survey, sleep difficulties were one of the top five symptoms that affected the daily activities of SSc patients [40]. In a cross-sectional study, the authors found that around 50% of SSc patients experienced a decline in the quality of their sleep [41]. Furthermore, a recently published study revealed that 84% of SSc patients demonstrated poor sleep quality, with 20% experiencing excessive daytime sleepiness [43]. Although numerous studies and comprehensive review articles have investigated the underlying causes of sleep disturbances in connective tissue diseases, none have explored the role of sleep hygiene [39–44]. To the best of our knowledge, there is no data in the literature on sleep hygiene and the relationship between sleep hygiene and sleep quality in patients with SSc.

The present study also exhibited that specific subscales of the PSQI, such as sleep quality, sleep latency, sleep efficiency, and sleep disturbances were more problematic in SSc patients. Sariyildiz et al. showed that SSc patients had significantly higher scores than healthy controls in certain domains of the PSQI, including sleep quality, sleep latency, sleep efficiency, sleep disturbance, and daytime dysfunction, and total PSQI scores [8]. A study by Figueiredo et al. found that SSc patients with sleep disturbances had higher scores in all domains than those without [9]. Similarly, a recently published study reported that patients with SSc exhibited longer sleep latency and reduced sleep efficiency compared to controls. Notably, this study was the first to demonstrate an association between electrolyte imbalance and disordered sleep in SSc patients. Specifically, lower serum potassium and iron levels were found to be associated with poorer sleep quality [45]. According to an editorial letter by Josef Finsterer, numerous intrinsic and extrinsic factors influencing sleep quality in general should be considered in the analysis. The author’s perspective is both valid and widely agreeable [46].

This study revealed that the lower quality of sleep in SSc was associated with pain, GERD, depressive symptoms, anxiety, and worse sleep hygiene. GERD was more common and NRS-pain scores were higher in SSc patients with poor sleep. We also showed that SSc patients with GERD exhibited 6.82 times higher odds of experiencing poor sleep. Similar to our study, numerous studies also defined gastrointestinal involvement, pain, and depression as independent predictors of sleep problems in SSc [4–6, 15, 41]. A study conducted by Horsley-Silva examining the impact of GERD on sleep quality found that SSc patients exhibiting GERD symptoms reported a more significant decrease in their sleep quality (almost 2.5-fold increase in the odds of poor sleep quality after adjusting age, gender, and BMI) [15]. Milette et al. contributed to the literature by showing that, in addition to the effect of gastrointestinal involvement and pain on sleep, pruritus might also be a reason for sleep issues in SSc [14]. In the existing literature, factors such as fatigue, digital ulcers, disease severity, skin involvement, disease duration, dyspnea, and compromised quality of life have been identified as variables associated with lower sleep quality in patients with SSc [5, 8, 9, 39, 41, 47–49]. However, we could not show any relationship between these last-mentioned clinical variables and sleep quality in our study.

The present study compared the results among three groups: SSc patients, RA patients, and healthy controls. We found that sleep hygiene was observed to be of poorer quality in patients with SSc compared to those with RA and HC. According to the PSQI, we showed that poor sleep was more common in SSc patients than in RA patients and HC and that SSc patients experienced 4.61 times more poor sleep when compared to RA patients and HC. In the literature, there is a scarcity of studies that compare sleep quality and clinical factors that can disrupt sleep quality between SSc patients and healthy individuals [8]. To the best of our knowledge, there is only one study that has explored the effect of clinical, autoimmune, and psychological factors on sleep disturbance in SSc and has compared these outcomes to patients with RA and healthy subjects [5]. According to that study, the authors found total PSQI score was higher in SSc patients than RA patients and HC. The specific subscales of PSQI, including sleep duration, sleep efficiency, and daytime dysfunction were significantly worse in patients with SSc [5].

This study also demonstrated that SHI scores were associated with fatigue, smoking cigarettes, decreased physical function and quality of life, depression, and anxiety. For each incremental increase of one unit in SHI scores, a concomitant decline was observed in sleep quality, sleep latency, sleep duration, daytime dysfunction, and total scores on the PSQI. We interpreted these results to mean that sleep hygiene plays an essential role in the sleep quality of patients with SSc, regardless of clinical variables associated with SSc. Therefore, sleep hygiene strategies that include many steps to increase sleep hygiene can ameliorate sleep disturbance in these patients. A recent study evaluating the effects of sleep hygiene education on sleep quality, pain, and depression in individuals with fibromyalgia demonstrated that sleep hygiene education effectively improved sleep quality and alleviated pain and depression [50]. However, there are no studies showing the efficacy of implementing sleep hygiene strategies for improving the sleep quality of SSc patients.

This study has some limitations. First, we did not use objective assessment tools to evaluate sleep hygiene, sleep quality, pain, fatigue, physical functioning and quality of life, depression, and anxiety. Instead, we performed instruments reliant on patient-reported measures for investigating these clinical variables. Second, the design of the study may have constraints on the data obtained from the participants. A prospective study can provide more comprehensive and detailed information. On the other hand, our study has also many strengths. Based on available knowledge, this study is the first to investigate sleep hygiene and its interplay with sleep quality in patients with SSc. In addition, the sample size is sufficient to make correct assumptions from the analyses. Finally, despite the lack of objective assessment tools, we employed tools that have been proven to be reliable and valid.

In conclusion, SSc patients tend to have lower sleep hygiene and worse sleep quality. Factors including fatigue, smoking, reduced physical function and quality of life, depression, and anxiety are closely associated with compromised sleep hygiene. Meanwhile, issues such as GERD and pain directly affect the quality of sleep. Poor sleep hygiene ultimately results in declining sleep quality for SSc patients. Consequently, enhancing sleep hygiene practices may serve as a crucial strategy to improve the overall sleep quality in SSc.

## Data Availability

Access to the data is restricted and can be obtained solely through the corresponding author upon reasonable request. The data is not publicly available due to the data protection legislation as the data contains information that could compromise the privacy of the research participants.
